# Supplementary figures and images for: Rigid-body fitting to atomic force microscopy images for inferring probe shape and biomolecular structure
Source: PLoS Comput Biol. 2021 Jul 20;17(7):e1009215. doi: 10.1371/journal.pcbi.1009215 (PMC8323932; doi:10.1371/journal.pcbi.1009215)

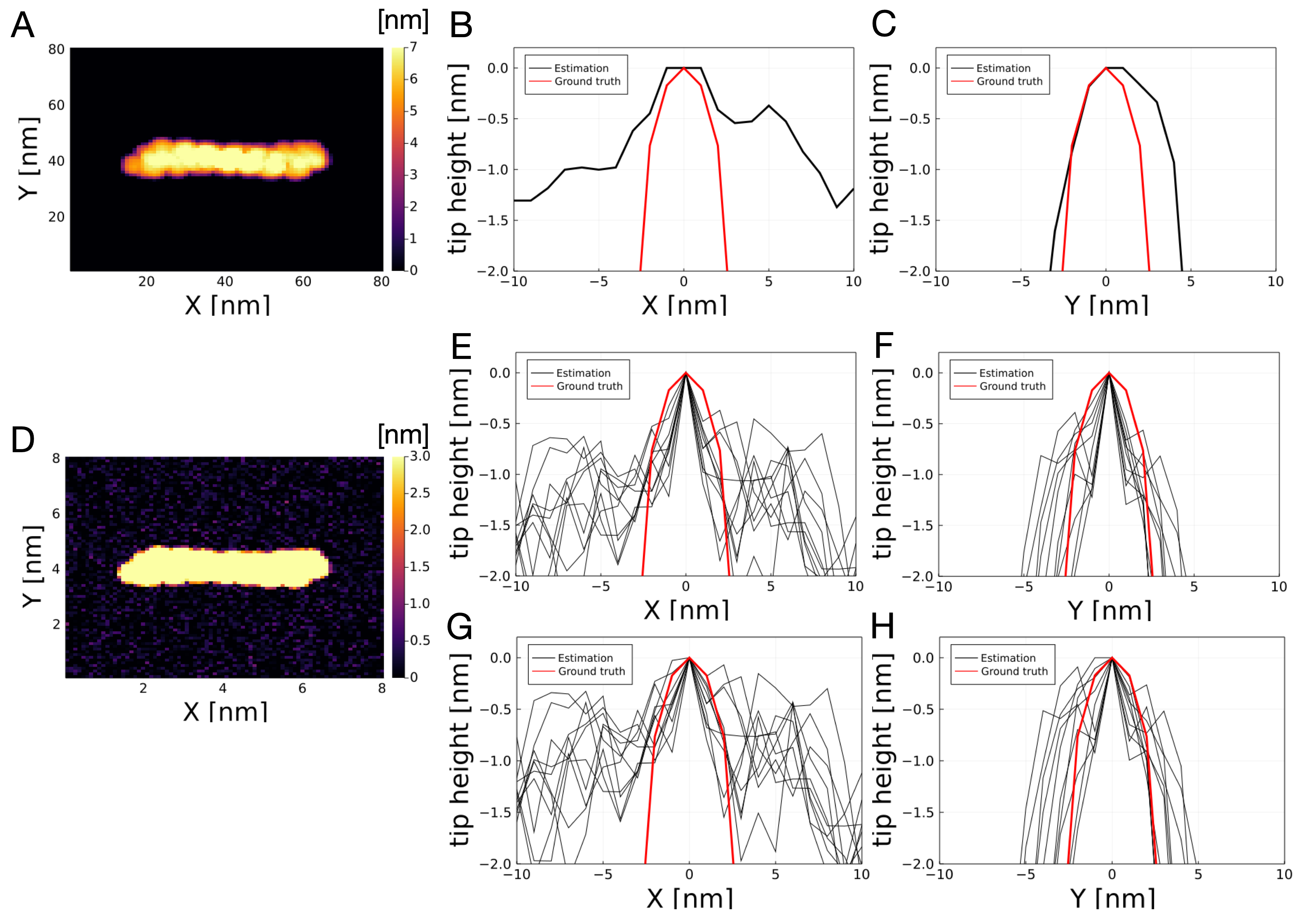

Supplement: S6 Fig — (A) Noise-free pseudo-AFM image of the 15-mer actin filament used in the twin-experiment in Fig 4. The image is generated by a 3 nm/20 degree probe, with the pixel width 1 nm. No noise is added to the image. (B) Cross sections of tip shapes along X-axis. The red line denotes the ground-truth of the tip shape, i.e., the tip shape used for generating the pseudo-AFM image. The black line is the estimation by the blind tip reconstruction algorithm. (C) Cross sections of tip shapes along Y-axis. (D) Pseudo-AFM image with Gaussian noise. Ten different images are generated by adding spatially independent Gaussian noise with the mean 0 nm and the standard deviation 0.3 nm. One of the ten images is shown. (E) Cross sections of tip shapes along X-axis. The red line denotes the ground-truth of the tip shape. The black lines show the estimated tip shapes by the blind tip reconstruction from the ten images using the threshold parameter of 0 nm. (F) Cross sections of tip shapes along Y-axis. (G) and (H) are estimated tip shapes by using the threshold parameter of 0.3 nm. (TIFF) [file pcbi.1009215.s006.tiff]

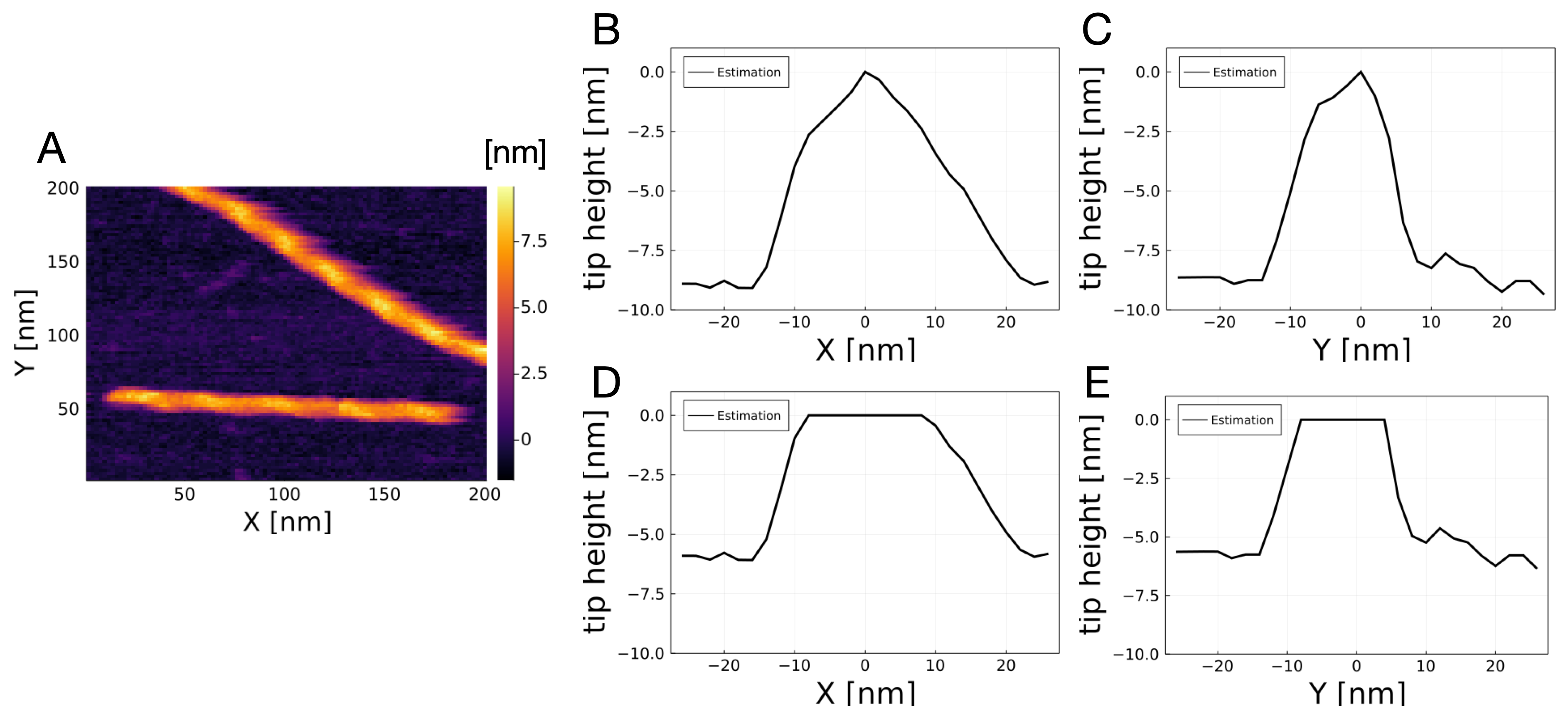

Supplement: S7 Fig — (A) The real AFM image of actin filament analyzed by the blind tip reconstruction. This is the same AFM data as those analyzed in Fig 5. The stage height is corrected by the fitted surface plane. (B) Cross section of the estimated tip shape along X-axis. Blind tip reconstruction is applied by using the threshold parameter of 0 nm. (C) Cross section along Y-axis. (D) and (E) are estimated tip shapes by using the threshold parameter of 0.3 nm. (TIFF) [file pcbi.1009215.s007.tiff]
